# Fecal estrogen, progestagen and glucocorticoid metabolites during the estrous cycle and pregnancy in the giant anteater (*Myrmecophaga tridactyla*): evidence for delayed implantation

**DOI:** 10.1186/1477-7827-11-83

**Published:** 2013-08-27

**Authors:** Katrina K Knott, Beth M Roberts, Morgan A Maly, Carrie K Vance, Jennifer DeBeachaump, Jackie Majors, Peter Riger, Heather DeCaluwe, Andrew J Kouba

**Affiliations:** 1 Department of Conservation and Research, Memphis Zoo, 2000 Prentiss Place, Memphis, Tennessee, USA; 2 Biochemistry, Molecular Biology, Entomology, and Plant Pathology, Mississippi State University, 32 Creelman Street, Mississippi, Mississippi State, USA; 3 Nashville Zoo, Grassmere, 3777 Nolensville Road, Nashville, Tennessee, USA; 4 Current Address: The Houston Zoo, 6200 Hermann Park Drive, Houston, Texas, USA

**Keywords:** Enzyme immunoassays, Fecal steroid hormones, Gestation, Non–invasive monitoring, Xenarthra

## Abstract

**Background:**

Declining numbers of wild giant anteaters highlight the importance of sustainable captive populations. Unfortunately, captive reproductive management is limited by the lack of external physical indicators of female reproductive status and the aggressive behavior of males. We examined the endocrinology of the estrous cycle and pregnancy, and whether delayed implantation is a gestational strategy for giant anteaters as described for other xenarthrans.

**Methods:**

Feces were collected from seven captive females 3–5 times weekly and mating was recorded. Concentrations of estrogen (estrone–glucuronide, E1, and estradiol–17β, E2), progestagen (20–oxo–progestagens, P4), and glucocorticoid (GC) metabolites were examined in fecal extracts by enzyme immunoassay.

**Results:**

Estrous cycles for nulliparous females (6 cycles, n = 2) compared to the multiparous female (6 cycles, n = 1) were shorter (47.3 +/− 4.3 days versus 62.5 +/− 2.6 days) with relatively lower luteal phase concentrations of P4 (49.4 +/− 2.9 ng/g versus 136.8 +/− 1.8 ng/g). The four remaining females had unclear ovarian activity: two females exhibited apparent luteal activity but unclear fluctuations in estrogens, while the other two females had parallel fecal P4 and estrogens concentrations. Pregnancy ranged 171–183 days with females returning to estrus post–partum as early as 60 days (n = 3, 1.8-4 years of age at mating). Delayed implantation was indicated by a biphasic elevation in fecal P4 metabolites: the initial 4–fold increase occurred for 81–105 days and was followed by a 26–fold secondary rise in P4 metabolites lasting 66–94 days prior to parturition. Fecal GC was correlated with fecal estrogens and greatest during estrus, late pregnancy, and six days prior to parturition (estrous cycle GC, 14.4-62.8 ng/g; pregnancy GC, 13.6-232.7 ng/g).

**Conclusions:**

Estrous cycles of giant anteaters occurred year–round, but were shorter and more intermittent in younger nulliparous animals compared to a multiparous female. A pronounced elevation in fecal P4, estrogen, and GC occurred during late gestation after an initial post-mating delay providing evidence for delayed implantation. Adrenocorticoid activity indicated impending parturition. Differences in estrous cycle characteristics with age and the protracted but variable gestation length must be considered to improve reproductive success and neonatal survival in giant anteaters.

## Background

The number of giant anteaters (*Myrmecophaga tridactyla*) endemic to Central and South America is currently in decline due to the transition of their grassland habitats into soja and sugar plantations, damage by fire and flooding, vehicle mortality, and hunting pressure [1,2]. As a result, giant anteater populations have been listed as decreasing in Appendix II of The Convention on International Trade in Endangered Species (CITES), and Near Threatened by the International Union for Conservation of Nature and Natural Resources (IUCN) [2-4]. This decline has led to a genetic bottleneck for the wild population [5]; consequentially, captive assurance colonies have become important for maintaining genetic diversity. In addition, captive populations can be used to educate the public about the unique biology of this species, raise awareness of *in situ* recovery efforts, and generate support for field conservation programs [6]. The long-term sustainability of the captive population is limited by high rates of first year mortality, ranging from 35-39% of births [4]. Because males are often aggressive towards females and young, one of the most significant challenges is safely introducing animals for breeding and removing males prior to parturition. Furthermore, overt estrus and pregnancy status is difficult to determine by animal care staff due to the lack of external physical indicators. Measurable physiological markers of sexual maturity, estrus, pregnancy, and impending parturition in giant anteaters are needed to improve reproductive success and neonatal survival.

Despite the fact that xenarthrans such as the giant anteater are considered the earliest of the placental mammals [7], few studies have described their reproductive biology (e.g., captive husbandry, reproductive behavior, placental phylogeny, reproductive endocrinology [2,4,8-10]). Giant anteaters live for approximately 15 years in the wild, but captive females can live beyond 28 years and successfully reproduce into their mid 20s [4]. Young animals grow rapidly, requiring at least three years to reach full adult size [9], yet in captivity 25% of recorded first births occurred in females 2–3 years of age [4]. Giant anteaters are polyestrous with reported breeding in the wild observed more often during the summer months (December in the Southern Hemisphere) [2,9]; nevertheless, births have been reported during every month of the year in both wild and captive populations [2,4,9]. To date there has been only a single paper available describing the reproductive endocrinology of the estrous cycle and gestation in giant anteaters [10]. While that report was an excellent initial examination, that study did not discuss the potential factors contributing to the variability in estrous cycle characteristics among individuals and was unable to examine endocrine profiles through a complete pregnancy. Therefore, questions remain regarding the reproductive endocrinology during sexual maturity and pregnancy, the seasonality of breeding, and whether giant anteaters exhibit delayed implantation (i.e., embryonic diapause). Variable breeding periods and gestation length have led many to hypothesize that giant anteaters exhibit a delayed implantation strategy similar to other xenarthrans, such as the nine-banded armadillo (*Dasypus novemcinctus*) [11,12]. However, evidence in support of delayed implantation in giant anteaters has yet to be described.

The purpose of this study was to examine the reproductive endocrinology of the estrous cycle and pregnancy in giant anteaters, and provide support for delayed implantation during gestation. The occurrence and duration of reproductive events were described based on changes in fecal estrogens (estrone–glucuronide, E1, and estradiol–17β, E2), progestagens (20–oxo–progestagens, P4), and glucocorticoid (GC) metabolites. This study expands upon the previous report of reproductive hormones in giant anteater [10] by examining the effect of age and parity on estrous cycle characteristics and provides evidence for delayed implantation during three complete pregnancies. In addition, this study provides an evaluation of adrenocorticoid activity during reproductive events. These data improve knowledge of giant anteater reproductive physiology, and provide guidelines for diagnosing pregnancy.

## Methods

### Animals and sample collection

There are currently 112 giant anteaters in U.S. zoos (about a 1:1 sex ratio) spread across 46 facilities. The giant anteater breeding facility at the Nashville Zoo (latitude, 36.1, longitude, -86.7) holds the largest number of animals within the U.S. This study was initiated as a result of inquiries from managers at the Nashville Zoo needing assistance in determining the reproductive cyclicity of the animals at their facility. Five of the females in this study (Emilia SB374, Lia SB376, Praim SB411, Odelia SB379, Gabriella SB395) were wild born at the rehabilitation/ breeding facility in Guyana, while the remaining two individuals (Monita and her daughter, Maripi) were born in captivity. The exact birth date for the wild born animals was unknown; therefore, the first day of the year (January 1) was used to estimate the age range during the sampling period (Table 1). Females were housed with a male, and had free access to both indoor and outdoor enclosures. Males were only removed prior to anticipated parturition based on changes in female behavior. Giant anteaters were fed a gruel mixture of Mazuri Leafeater and Mazuri Insectivore diets (1:1 v/v water; http://www.mazuri.com) and had access to water throughout the day. Mating behavior was observed opportunistically and parturition dates were recorded by Nashville Zoo staff. ‘Wrestling’ periods observed by keepers were also considered to be possible matings since breeding in giant anteaters can occur within minutes and breeding behaviors could have been mistaken as ‘play’ behaviors in young animals. One female (Monita) had two pregnancies prior to the sampling period (9/2001; 1/2003–Maripi), whereas three females (Emelia, Lia, Praim) gave birth to their first young during the study period. Offspring were kept with females and allowed to nurse naturally. All offspring survived after birth to first year. Fecal samples were collected 30–90 minutes after defecation during the day or from an overnight sample, approximately three times per week. Fecal samples were stored at −20°C and transferred to the Memphis Zoo Conservation and Research Department for subsequent extraction of fecal hormone metabolites and enzyme immunoassays. Since this study involved only the non-invasive collection of feces, the approval of an ethics committee at either the Memphis Zoo or Nashville Zoo was not required.

**Table 1:** Reproductive characteristics of giant anteaters

**Animal name**	**Studbook number**	**Age range (years)**	**Estrous cycles (count)**	**Estrous cycle (days)**	**Luteal phase (days)**	**Pregnancy (days)**	**Post-partum return to estrous (days)**
Monita	314	6.2 – 7.8	6	62.5 ± 2.6 (55 – 74)	24.8 ±1.3 (22 – 33)		
Emelia	374	3.6 – 5.6	3	47.3 ± 4.3 (42 – 56)	25.2 ± 0.7 (23 – 27)	183	151
Lia	376	3.5 – 5.6	3	49.3 ± 2.8 (46 – 55)	20.1 ± 0.4 (19 – 22)	171	60
Odelia	379	3.5 – 3.7	0		16, 16		
Gabriella	395	1.3 – 2.9	0				
Praim	411	1.5 – 2.6	0		16, 28	175	88
Maripi	402	0.9 – 1.4	0				

Data are shown as the mean ± SEM (range). Age range was estimated from beginning to end of the fecal collection period. Although two luteal phases were observed for Odelia and Praim, the lack of definitive E peaks did not allow for an estimation of total estrous cycle length. Gabriella and Maripi did not exhibit luteal activity during the study period.

### Extraction techniques

Four extraction techniques were compared using 0.503 ± 0.001 g of wet feces from five randomly selected sampling days. For the Phosphate Method, thawed fecal samples were solvated with 5 ml of a modified phosphate buffer solution (100 mM PBS, pH 7.0, 0.1% BSA, 5% Tween 20, 20% methanol), vortexed for 24 hours, and the supernatant collected for analysis [13]. For the 80% Ethanol Method, thawed fecal samples were solvated overnight in 5 mL of 80% ethanol for 20 hours and the supernatant was diluted 1:1 (v/v) with enzyme immunoassay buffer (0.2 NaH_2_PO_4_, 0.2 M Na_2_HPO_4_, pH 7, 0.1% BSA) following procedures previously performed at the Memphis Zoo for tamandua and aardvark (data unpublished). For the 40% Methanol Method, thawed fecal samples were solvated in 5 mL of 40% methanol and the supernatant was diluted with phosphate buffer (40 mM PBS, 0.1% BSA, pH 7.2) as previously used for bonobos [14]. For the Petroleum Ether: Methanol Method, thawed fecal samples were solvated in 5 mL of 80% methanol, vortexed with petroleum ether and methanol (1:3 v/v), centrifuged, and the supernatant diluted 1:1 (v/v) with Tris buffer (20 mM Tris buffer, 30 mM NaCl, 0.1% BSA and 0.1% Tween 80; pH 7.5) as previously described by Patzl et al. [10] for giant anteaters. Serial dilutions of each sample extract (1:2, 1:4, 1:8, and 1:16 with EIA buffer) were prepared to determine which extraction method produced the highest concentration of fecal hormone metabolites and whether hormone metabolites were bound to antibodies in a dose–dependent manner under our assay conditions (Additional file 1: Figure S1). As a result of this preliminary investigation, we used the 80% Ethanol extraction method for the remaining samples to eliminate the use of strong solvents and because this method yielded the greatest concentrations of hormones within the optimum optical density (OD) range (final dilutions of 1:4 and 1:8 within 0.3 to 0.6 OD) under the assay conditions in our laboratory.

### Enzyme immunoassays

Concentrations of immunoreactive metabolites (progestagens [P4], estradiol [E2], estrone–glucuronide [E1], and glucocorticoids [GC]) in fecal extracts were determined using a single antibody competitive enzyme immunoassay (EIA) as previously described [13,15]. Briefly, polystyrene 96–well microtiter plates (NUNC plates, Thermo Scientific, Rochester, NY) were coated with antibody and stored overnight at 4°C. Fecal extracts were diluted in enzyme immunoassay buffer (EIA buffer; 0.1 M phosphate–buffered saline containing 0.1% bovine serum albumin) prior to assay development to ensure 20–80% total binding. Standards, controls and diluted fecal extracts were run in triplicate. Hormone conjugated to horseradish peroxidase (HRP) was applied to compete for binding sites on the antibodies, and plates were allowed to equilibrate at room temperature for two hours. Azino–bis–3–ethyl benzthiazoline–6–sulfonic acid (40 mM, ABTS) was used as the substrate and hydrogen peroxide as the catalyst to detect the percent of hormone specific–HRP conjugate bound to the antibody using a MRX Revelation microplate reader (ThermoScientific, Rochester, NY). Optical densities were read at 405 nm. The concentrations of fecal hormone metabolites were determined by the inverse of the bound fraction as compared to a standard curve. To limit the interference of ethanol to assay sensitivity for GC, samples requiring dilutions of less than 1:5 of fecal extract under the assay conditions were dried by air and reconstituted with EIA buffer.

All antibodies and hormone–HRP conjugates were provided by C. Munro and the Clinical Endocrinology Laboratory at the University of California Davis. The P4 assay employed a rabbit anti–4–progesterone–11–ol–3, 20–dione–BSA (CL425) monoclonal antibody (1:6000) and progesterone–3CMO–HRP conjugate (1:60000) as described previously by Graham et al. [16]. The P4 antibody cross–reacts with 100% progesterone (4–pregnen–3,20–dione), 188% 4–pregnen–3α–ol–20–one, 172% 4–pregnen–3β–ol–20–one, 147% 4–pregnen–11α–ol–3,20–dione, 94% 5α–pregnen–11–3β–ol–20–one, 64% 5α–pregnan–3α–ol–20–one, 55% 5α–pregnane–3,20–dione, 12.5% 5β–pregnane–3β–ol–20–one, and <10% for all other tested steroids [16]. Progesterone (4–pregnen–3,20–dione, Sigma–Aldrich, St. Louis, MI) was used as the standard in concentrations ranging from 0.244–500 pg/well. The E2 assay employed a rabbit anti–17β–estradiol (R4972) polyclonal antibody (1:10000) and estradiol–3CMO–HRP conjugate (1:25000). The E2 antibody cross–reacts with 100% estradiol, 3.3% estrone, and < 1% with for all other tested steroids. Estradiol (Sigma–Aldrich, St. Louis, MI) was used as the standard in concentrations ranging from 0.975–2000 pg/well. The E1 assay employed a rabbit anti–estrone–3–glucuronide (R522-2) antibody (1: 25000) and estrone–3CMO–HRP conjugate (1:50000). The E1G antibody cross–reacts with 100% estrone–3–glucuronide, 66.6% estrone–3–sulfate, 23.8% estrone, 7.8% estradiol–17β, 3.8% estradiol–3–glucuronide, 3.3% estradiol–3–sulfate, and < 1% of all other tested steroids [15]. Estrone 3(β–D–glucuronide) (Sigma–Aldrich, St. Louis, MI) was used as the standard in concentrations ranging from 0.785-400 pg/well. The glucocorticoid assay employed a poly–clonal rabbit anti–corticosterone (CJM006) antibody (1:20000) and corticosterone–3CMO–HRP conjugate (1:90000) following Metrione and Harder [17]. The corticosterone antibody cross–reacts with 100% corticosterone, 14.25% desoxycorticosterone, 2.65% progesterone, 0.23% cortisol, and < 1% of other tested steroids. Standard concentrations ranged from 0.244-2000 pg/well.

Serial dilutions of fecal extracts were parallel to standard curves for all four assays examined (Additional file 2: Figure S2). Intra–assay coefficients of variation for low (~25% total binding) and high (~80% total binding) controls were < 10% for all hormone assays. Inter–assay coefficients of variation of low and high controls were 19%, 12%, 19%, and 10%, for P4, E2, E1, and GC respectively. Recoveries of standard hormone concentrations added to pooled fecal extract were: P4, y = 0.802x–0.299, r^2^ = 0.999; E1, y = 0.703x + 0.534, r^2^ = 0.999; E2, y = 1.022x–1.97, r^2^ = 0.998; GC, y = 0.854x + 3.637 r^2^ = 0.999. The concentrations of hormone metabolites in feces were reported as ng/g wet feces.

### Data analysis

Reproductive phases were determined as previously described [10,18,19]. The length of an estrous cycle was measured by the number of days between estrogen (either E1 or E2) peaks. The length of the non–pregnant luteal phase was determined by the number of days between the estrogen peaks to the first day that P4 concentrations returned to baseline (baseline Mean ± 2 SD) for two or more consecutive sampling days [18]. Basal E1 and E2 values were calculated from the luteal phase (Mean ± 2 SD). The initial elevation in P4 during pregnancy was established as the point when P4 concentrations rose 2 SD above basal P4 for two or more consecutive sampling days [18]. The end of the primary and beginning of the secondary rise was determined to be when P4 concentrations increased 2 SD above primary concentrations for two consecutive days. The secondary rise ended at parturition. The beginning of primary rise in P4 through parturition date determined the total duration of pregnancy. These rules were used for calculation of the average concentrations of hormones during each phase or reproductive event. For statistical descriptions and comparisons, estrous cycles were lined up to estrogen peaks (Day 0) and pregnancies were aligned by parturition date (Day 0). Data are presented as Means ± SEM.

Normality of the data was examined using the Shapiro–Wilks test. Significant differences for non-parametric data were assessed using the Mann–Whitney test for two groups (e.g., basal and peak estrogens) and the Kruskal–Wallis One Way Analysis of Variance for multiple comparisons. The Dunn’s test was used to assess the differences between pairwise multiple comparisons. Spearman’s correlation was used to assess the relationship between endocrine measures. For data that was normally distributed (e.g., extraction comparisons), the differences between groups were examined by Analysis of Variance (ANOVA) with Tukey’s adjustments for multiple comparisons. Linear regression was also used to assess the relationship between dilution factors and concentration of immunoreactive metabolites of endogenous steroids in each extraction method. Significance was considered to be p < 0.05. Microsoft Excel 2007 was used for data iterations, and Sigma Plot 12.0 was used for statistical analyses.

## Results

### Extraction comparisons

Giant anteater feces extracted using the 80% Ethanol, 40% Methanol, and Petroleum Ether:Methanol Methods provided similar concentrations of E1, E2 and P4 metabolites (Additional file 1: Figure S1) and serial dilutions produced significantly linear results (p < 0.01). The Phosphate Method was inferior to the other extraction methods tested as determined by the lower concentrations of P4 metabolites at the 1:2 dilution, and the non–linearity of immunoreactive metabolites determined in the E1 assay (p = 0.109).

### Biological validation

**Figure 1:**
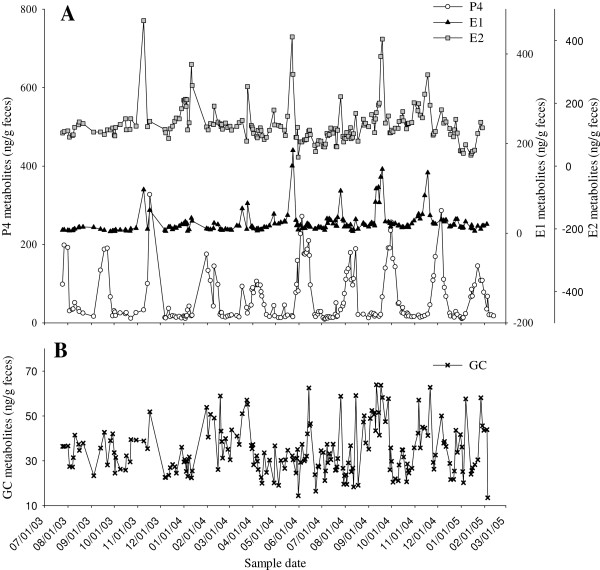
**Fecal hormone metabolites profile in an adult female. (A)** Profile of fecal P4, E1, and E2 metabolites over a 20 month period in an adult female giant anteater (Monita, SB314) after two consecutive pregnancies (prior parturition date of 1/25/03). **(B)** Profile of fecal GC metabolites in the same female giant anteater during estrous cycling.

**Figure 2:**
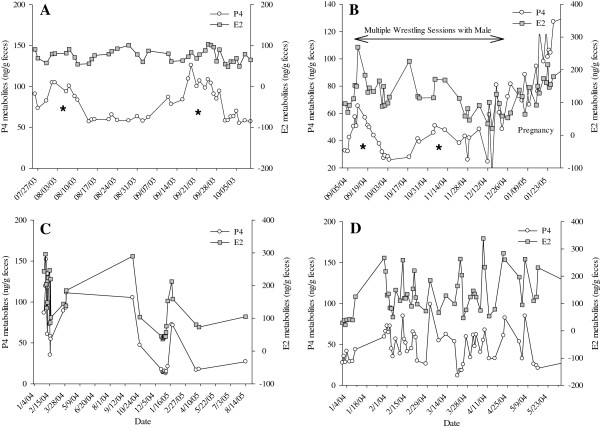
**Concentration of fecal P4 and E2 metabolites in females with indeterminate estrous cycles. (A)** Odelia **(B)** Praim, was pregnant during the study period; **(C)** Gabriella; **(D)** Maripi. Asterisk indicate luteal activity as determined by elevated P4 metabolites for ~20 days. Note the parallel concentrations of P4 and E2 metabolites in **C** and **D**, and the apparent luteal activity (marked) but lack of definite E2 peaks in females represented in **A** and **B**.

**Figure 3:**
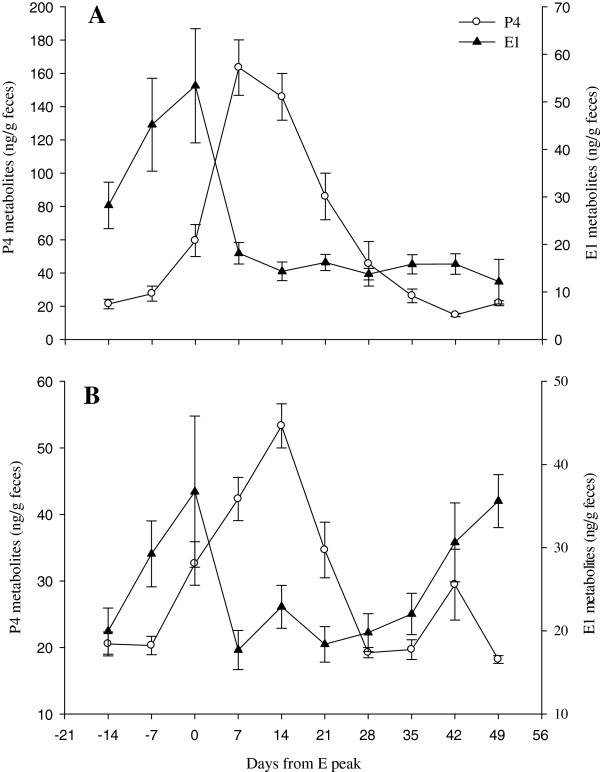
**Fecal hormone metabolites during the estrous cycle of female giant anteaters.** Mean weekly concentrations of fecal P4 and E1 metabolites during the estrous cycles: **(A)** a multiparous female (Monita: n = 6 cycles), and **(B)** two nulliparous females (Emelia and Lia: n = 6 cycles). Data are reported as mean ± SEM each week relative to the peak E1 concentration during estrus. Note the shorter estrous cycle length (**B**, mean, 47 days) and the three-fold lower concentrations of P4 metabolites in the nulliparous females relative to the multiparous female (**A**, mean estrous cycle length, 62 days).

**Figure 4:**
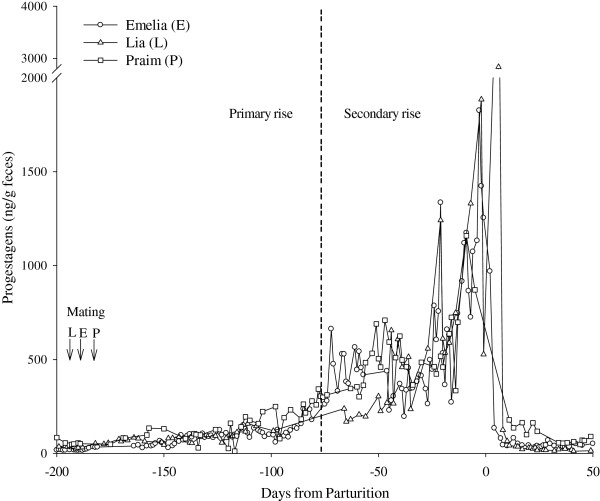
**Fecal progestagen metabolites during pregnancy.** Primary and secondary rise of fecal progestagens (P4) in three female giant anteaters (Emelia, E; Lia, L; Praim, P) during pregnancy. The dashed line indicates a mean demarcation (−82 days) between the primary and secondary rise in fecal P4 for the three females.

Concentrations of estrogens determined using E1 and E2 immunoassays were correlated (r = 0.538, p < 0.0001, n = 178; Figure 1). Estrogen peaks were significantly greater than baseline values (E1, p < 0.001; E2, p < 0.001, n = 12 cycles), but the magnitude of this difference was greater for E1 metabolites (peaks about 4‒fold over basal) than E2 metabolites (peaks 2.7‒fold over baseline; Table 2). An estrogen peak as determined by both E1 and E2 immunoassays occurred within eight days of behavioral estrous (i.e. recorded mating) for two of the three pregnant females. The youngest pregnant animal in the study (Praim) showed two luteal phases prior to pregnancy and the second estrogen peak occurring during this period was believed to correspond to behavioral estrus, as this female was involved in multiple wrestling sessions with a male during this time period (Figure 2B). The selected immunoassay for determination of P4 metabolites proved to be an adequate marker of the different phases of the reproductive cycle based on a significant increase in P4 above baseline values during the luteal phase (2.7-7.1 fold above baseline for Monita, Emelia, and Lia, p < 0.05, Table 2, Figure 3). Moreover, a primary rise in P4 during the first half of pregnancy (3.0‒5.4 fold increase above baseline) and a secondary rise in P4 during the latter half of pregnancy (20.1-34.9 fold increase above basal levels) was observed (p < 0.05, Table 2 Figure 4). The use of fecal GC analyses as a marker of adrenocortical activity was also validated in this study as evidenced by elevated GC concentrations during the acute stressful periods of mating and parturition.

**Table 2:** Comparison of fecal hormone metabolites in female giant anteaters

	**Animal ID**			
	**Monita**	**Emelia**	**Lia**	**Praim**
**P4 metabolites**				
**Basal**	19.4 ± 1.8	15.5 ± 2.3	20.9 ± 1.3	ND
**Luteal phase**	136.8 ± 1.8	41.2 ± 5.1	57.5 ± 0.7	ND
**Pregnancy, primary rise**	ND	83.2 ± 4.3	62.2 ± 3.9	155.6 ± 16.6
**Pregnancy, secondary rise**	ND	540.6 ± 43.1	419.5 ± 61.1	467.0 ± 39.4
**E1 metabolites**				
**Basal**	16.8 ± 1.1^a^	32.6 ± 8.0^b^	8.9 ± 1.4^c^	ND
**Peak estrus**	96.3 ± 20.3	121.1 ± 43.3	27.7 ± 7.1	15.6 (1 peak)
**Pregnancy**	ND	103.8 ± 6.4^a^	53.6 ± 5.1^b^	53.8 ± 6.7^b^
**E2 metabolites**				
**Basal**	114.4 ± 3.7^a^	104.9 ± 5.9^a^	69.8 ± 7.5^b^	ND
**Peak estrus**	298.3 ± 36.3	283.6 ± 100.7	177.0 ± 28.8	112.0 (1 peak)
**Pregnancy**	ND	204.5 ± 11.4^a^	151.6 ± 11.3^b^	210.5 ± 16.9^ab^
**GC metabolites**				
**Estrous cycle**	33.5 ± 0.9	ND	ND	ND
**Pregnancy, primary rise**	ND	52.7 ± 2.3^a^	33.2 ± 2.4^b^	36.8 ± 2.3^b^
**Pregnancy, secondary rise**	ND	70.4 ± 3.1^a^	90.0 ± 8.9^b^	55.8 ± 2.5^a^

Data are shown as the mean ± SEM (ng/g). Monita: adult, n = 6 estrous cycles, no pregnancy during the study period; Emelia and Lia, 1 pregnancy; Praim, n = 0 observed estrous cycles; 1 pregnancy. ND = not determined in this study. Different letters indicated significant differences (p < 0.05) between animals for the corresponding parameter.

### Estrous cycle characteristics

Estrous cycle length, as determined by the interval between estrogen peaks, ranged from 42 to 74 days (Table 1). The length of the estrous cycles for the multiparous female (Monita) were 14 days longer than those for nulliparous females Emelia and Lia (Table 1; Figure 3; p < 0.05) and non-distinct in the remaining 4 females examined. As such, the female giant anteaters from this study could be divided into four estrous cycle categories: (1) a multiparous female (Monita) with regular estrous cycle length (mean, 62.5 days; range, 55-74 days) with fecal P4 concentration during the luteal phase ranging 21-286 ng/g (mean, 136.8 ng/g; Tables 1 and 2, Figure 3A); (2) nulliparous females (Emelia, Lia) with shorter estrous cycle lengths (mean, 47.3 days; range, 42-56 days) than the multiparous female and comparatively lower luteal phase concentrations of P4 (mean, 41.2 and 57.5 ng/g; range, 16–143 ng/g feces; Tables 1 and 2, Figure 3B); (3) nulliparous females (Praim, Odelia) with unclear estrogen peaks but apparent increase in fecal P4 (mean, 73 ng/g; range, 42.4-125.9 ng/g; Figure 2A and B) suggesting ovulation/luteal phase; and (4) non-cycling females with parallel concentrations of fecal P4 and E2 metabolites (Figure 2C and D). Monita was the only captive–born cycling female examined and showed no apparent cycling irregularity related to season (Figure 1), whereas the wild–born females (Emelia, Lia) exhibited irregular cycling during July–December (data not shown).

### Evidence for delayed implantation during gestation

**Figure 5:**
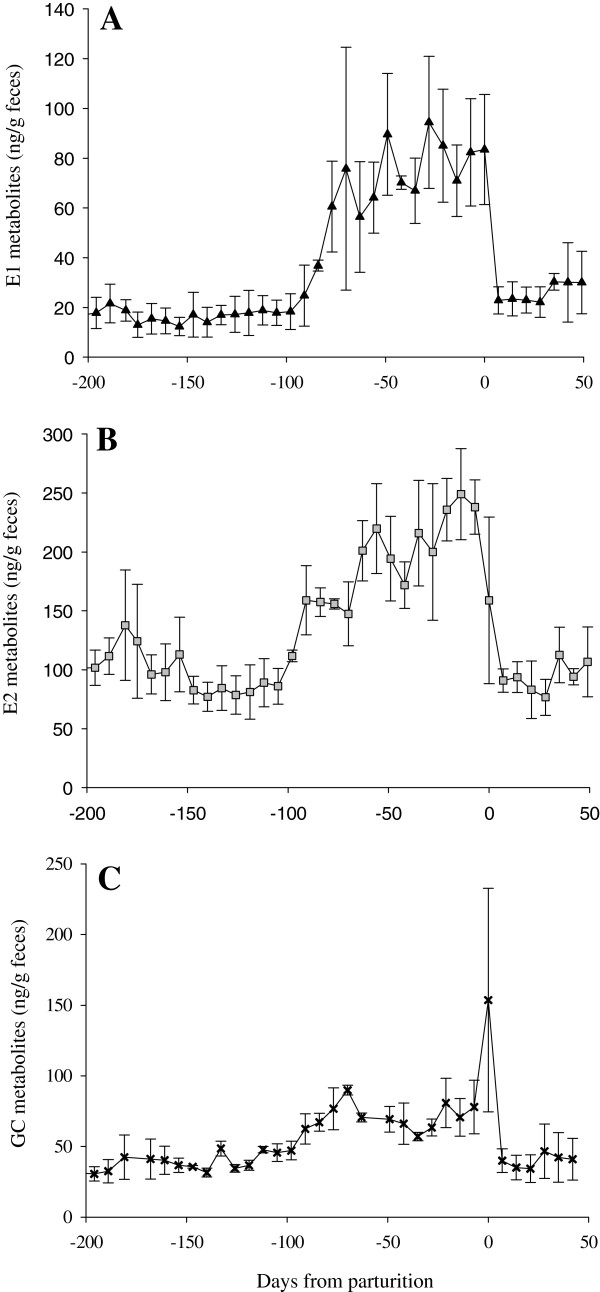
**Fecal estrogen and glucocorticoid metabolites during pregnancy.** Profile of **(A)** E1 metabolites, **(B)** E2 metabolites, and **(C)** GC metabolites in the feces of three female giant anteaters during pregnancy. Concentrations are reported as the average ± SEM during each week of the pregnancy.

Based on observed behavior, peak estrogens and changes in P4, Praim and Emelia bred and conceived in October and January, respectively, with parturition occurring during April and July. Conversely, Lia bred in July and birthed offspring in January. Gestation ranged from 171 to 183 days (n = 3) from the rise in P4 following breeding through parturition. A biphasic increase in fecal P4 metabolites was observed during all pregnancies (Figure 4). The first rise in fecal P4 lasted between 81 to 105 days (Emelia, 97 days; Lia, 105 days; Praim, 81 days) with concentrations elevated 4 fold (p < 0.05) above baseline for Emilia and Lia. A secondary increase in P4 concentration lasted 66 to 94 days (Emelia, 86 days; Lia, 66 days; Praim, 94 days) with concentrations 26 fold greater (p < 0.05) than baseline (Table 2; Figure 4). Metabolite concentrations of E1 and E2 were greatest during late pregnancy coincident with the secondary rise in P4 (Table 2; Figure 5A and B). Concentrations of P4, E1, and E2 remained elevated for 4 to 11 days after parturition. Reinstatement of estrous post–partum was similar for 2 of 3 pregnant animals (Lia, 60 days; Praim, 88 days). Concentrations of P4 and E metabolites remained elevated for a four-month period after parturition (but below pregnancy concentrations) in the remaining female, which resulted in an extended delay in post–partum estrus (Emelia, 151 days; Table 1).

### Glucocorticoid metabolite concentrations during the estrous cycle and pregnancy

Fecal GC metabolites varied over 4‒fold during the estrous cycles, and concentrations were not significantly different between estrous cycles (p = 0.121; Figure 1B). Fecal GC metabolite concentrations during estrous cycles were positively correlated to all steroids examined (p < 0.05), but this relationship was strongest between GC and E1 metabolites during cycles with well–defined estrogen peaks and luteal phases (p < 0.001, Figure 1).

Fecal GC metabolite concentrations were greater during pregnancy than during the estrous cycle, with the greatest elevation occurring late in pregnancy (p < 0.001; Table 2; Figure 5C). The highest fecal GC values for 2 of 3 pregnant females occurred on or near the day of the secondary P4 rise and ± 6 days of parturition (Figure 5C).The highest GC values for the remaining pregnant female (Praim) occurred 34-45 days prior to parturition.

## Discussion

The non–invasive study of fecal hormone metabolites determined by enzyme immunoassay has become a useful tool for monitoring the reproductive status of animals in captivity and in the wild. Sampling frequency and duration of fecal collections for endocrine studies must take into account anticipated reproductive events (e.g., sexual maturation, estrous cycling, and pregnancy) paired with observational information and knowledge of the animal’s reproductive biology [20]. In this study, fecal samples were serially collected from a multiparous female and six females reaching sexual maturity at < 4 years of age. This study greatly expands upon a previous report of fecal hormones in giant anteaters by Patzl et al. [10], which initially characterized estrous cycle and parturition length. Here, we are able to provide additional information of giant anteater reproduction through the biological validation of fecal hormone analyses, describe differences in estrous cycle characteristics by age and parity, confirm that sexual maturation can occur in as little as 1.8 years of age prior to overt cycling, show that there is no apparent seasonality to breeding in captivity, and provide a longitudinal evaluation of adrenocorticoid activity during the estrous cycle and pregnancy. In addition, this study is the first to provide endocrine evidence for a delayed implantation strategy during gestation in giant anteaters. Information on extraction and assay methodology are also provided as a guide for captive managers in the use of fecal hormone analyses to non-invasively monitor the estrous cycle and diagnose pregnancy in giant anteaters.

Extraction methods that utilized a high percentage of apolar solvents (e.g., ethanol and methanol) provided similar concentrations of steroid hormone metabolites from feces of giant anteater. Among the methods examined, the 80% Ethanol Method was selected as the best method because it involved the fewest number of steps and provided the highest concentration of P4, E1, and E2 metabolites. The Petroleum Ether: Methanol extraction used by Patzl et al. [10] for giant anteater was also examined in the present study and gave statistically similar results as the 80% Ethanol Method. Extraction procedures using a high percentage of apolar alcohols also yielded a better recovery of steroids from feces of livestock and felids [21,22]. The utility of extraction methods containing high concentrations of apolar solvents is based on the hydrophobic nature of the steroid hormone backbone and the polarity of side chains (e.g. hydroxyl, methyl, aromatic, etc.) and conjugates (e.g. sulfates and glucuronides) added during hormone synthesis, metabolism and excretion processes [23]. Determination of the best method for extracting steroid metabolites from feces of giant anteaters could be improved by radiolabeled hormone studies similar to those performed in other species [22,24]. Radiolabeled studies, however, can be difficult in non–tractable animals in which multiple fecal collections are difficult, or when laboratory space and equipment for use of radioactive materials are unavailable. Thus, the comparison of fecal hormone metabolite concentrations among different extraction methods is a useful way to easily determine which procedure is most applicable for the species being examined [20].

The selected antibody clone: HRP enzyme immunoassay systems described in this study are commonly used in zoological institutions for reproductive monitoring, and here we show they are also applicable for use in giant anteaters. Patterns of fecal estrogen, P4, and GC metabolite concentrations in female giant anteaters matched the observed and anticipated reproductive events, thus validating the use of these immunoassay systems. For example, estrogen peaks, determined using both E1 and E2 immunoassays, were observed within eight days of mating. Furthermore, the occurrence and magnitude of elevated fecal P4 metabolite concentrations were consistent with the luteal phase and pregnancy. The 3‒fold greater concentration of E2 versus E1 metabolites in giant anteater feces was consistent with the larger peak of ^3^H–estradiol–17β than ^3^H–estrone previously observed by Patzl et al. [10] and their conclusion that estradiol–17β was the major estrogen metabolite excreted. Our finding that concentrations of E1 and E2 metabolites were significantly correlated suggests that the proportion of metabolites excreted may vary little during the follicular phase and pregnancy. Patzl et al. [10] reported that progestagen metabolites in giant anteater feces included both 20α–OH– and 20–oxo progestagens, which they examined using antibodies against pregnane–glucuronide and progesterone, respectively. Profiles in giant anteater were similar with both immunoassay systems, but Patzl et al. [10] only showed data for 20α–OH– progestagens. This study is the first to report concentrations and profiles of 20–oxo–progestagens and suggest that this group-specific antibody (anti–progesterone monoclonal antibody, CL425), which has been proven to be useful for non–invasive monitoring of ovarian function in a variety of species [16,25], is also appropriate for defining and contrasting the non-pregnant and pregnant luteal phase in giant anteaters.

Although hormone concentrations and lengths of estrous cycles for giant anteaters reported in this study were similar to previous reports of xenarthrans ([10]; Table 3), we also report here that estrous cycle lengths of younger nulliparous females were 14 days shorter and P4 metabolite concentrations over 2-fold lower than those of a multiparous female. In this study, the multiparous female exhibited the anticipated polyestrous profile of giant anteaters as exhibited by regularly spaced E peaks occurring throughout the year. Each E peak was followed by an elevated concentration of P4 confirming ovulation and formation of functional corpus lutea. In contrast, younger nulliparous females had more intermittent and shorter estrous cycles or endocrine profiles that exhibited unclear ovarian activity (e.g., luteal activity without an observed estrogen peak or parallel steroid hormone profiles). All females were housed with a male for the duration of the study except when parturition was expected, and keepers also shifted males to find optimal pairings. At the Nashville Zoo, managers reported that after females were paired successfully with a male (i.e., living with each other without confrontation) they become quite bonded and would sleep together in the same kennels (P. Riger, personal communication). This bond may have been stronger for the multiparous female as she was older, had been at the facility for a longer period of time, and thus more accustomed to the presence of males. Although copulation was not observed, it is still unknown whether olfactory, tactile, or visual cues brought about by the presence of the male are required for ovulation in giant anteaters. Thus, irregular holding or shifting of males could explain the variable estrous cycle patterns and longer return to estrus post-partum in some females. Seasonality of breeding in wild populations is an evolutionary process where individual species react to weather, food security, habitat changes, and the availability of mates. In a zoological setting, food, habitat and general safety variables are taken away and animals are held in an optimal setting all year long excluding the need for seasonal breeding (and parturition date). Variation in the ovarian activity of nulliparous females in this study may also have been a result of their relatively younger age. Age at first reproduction for the majority of captive giant anteaters ranges 2-4 years [3,4,26], although a single female giant anteater was reported to give birth at 1.6 years [1,2]. The youngest pregnant female in this study was 1.8 years of age and this female did not exhibit clear ovarian cycling prior to breeding. Two other females in this study (Maripi and Gabriella) were similar or older in age (< 3 years) and considered to be non-cyclic based on parallel concentrations of steroid hormones. The few animals available for study did not allow for further examination of ovulatory cues and sexual maturation, but these topics are of great importance in reproductive management of giant anteaters and require further investigation.

**Table 3:** **Reproductive characteristics of giant anteaters ( *M. tridactyla *) and tamandua ( *T. tetradactyla *)**

**Species**	**Age range (years)**	**Estrous cycles (count)**	**Estrous cycles (days)**	**Luteal phase (days)**	**Pregnancy (days)**	**Post-partum return to estrus (days)**	**Citation**
*M. tridactyla*	0.5 – 7.8	12	55.4 ± 2.7 (42–74)	24.1 ± 1.1 (19 – 35)	176.0 ± 3.5 (171 – 183)	99.7 ± 26.9 (60 – 151)	This study^a^
*M. tridactyla*	< 2 – 13	10	51.4 ± 5.6 (44 – 63)	14 - 21	184	46.0 ±12.0 (28 – 70)	Patzl et al. 1998 [10]^a^
*T. tetradactyla*	3	6	44.3 ± 4.5 (39 – 50)	na	165	22	Kusuda et al. 2011 [27]^b^
*T. tetradactyla*	na	11	42.0 ± 3.0 (38 – 46)	21.3 ± 0.4	na	na	Hay et al. 1994 [19]^c^

Data are shown as the mean ± SEM (range) of all animals examined in each study. na = data not reported in the cited manuscript. ^a^EIA performed on fecal extractions; ^b^EIA performed on serum samples; ^c^EIA performed on urine samples.

Females in our study had similar gestational lengths as previously reported for giant anteaters and longer than reported for tamandua [1,3,10,27] (Table 3). By examining three complete pregnancies, we were able to confirm that elevated concentrations of fecal estrogens and P4 occured only during the late gestational period as previously summarized by Patzl et al. [10]. In addition, we were able to show that concentrations of GC are elevated during the late pregnancy. This endocrine profile supports the hypothesis that giant anteaters exhibit obligate diapause during pregnancy; an attribute that has not been previously described in this species, but has been reported in xenarthrans (nine–banded armadillo *Dasypus novemcinctus*), mustelids (mink *Mustela vison;* European badger *Meles meles*; spotted skunk *Spilogale putorius latifrons*) and ursids (giant panda *Ailuropoda melanoleuca*; polar bear *Ursus maritimus*; Andean spectacled bear *Tremarctos ornatus)*[12,28-33]. Obligate diapause occurs during every pregnancy converse to facultative diapause that only occurs in response to lactational or metabolic stress [11,31]. Obligate diapause is thought to allow the female to time parturition with environmental and/or endogenous conditions favorable for neonatal survival, and may or may not be tied to season [29]. Parturition apparently is not tied to season in giant anteaters as birth dates have been reported throughout the year in both wild and captive populations [2,4,9]. Therefore, it is not unexpected that 2 of the females in this study gave birth during July/April, and 1 female gave birth in January. As described above, variable parturition dates may also be an expected result for females held under the optimal captive environment. During diapause, the embryo remains at an arrested state until a yet unknown environmental and/or physiological signal triggers further embryonic development [29,31]. Changes in photoperiod, temperature, and maternal nutrient supply have been suggested as triggers to initiate implantation of the blastocyst as a result of the greater production of prolactin, estrogens, and modification of specific uterine proteins [29]. These changes also initiate a secondary surge in progestagens during late pregnancy by luteal cells, the feto–placental unit, or another extra–gonadal source following implantation [30,34]. Elevation of fecal estrogens during late pregnancy in xenarthrans and other species also supports fetal development, and is the result of greater aromatase activity in the corpus lutea and the placenta, as well as the result of increased hepatic clearance of estrogens prior to fecal excretion [10,13,27,34,35]. Fecal estrogens and progestagens returned to baseline levels within 11 days of parturition, and estrous cycling post–partum was observed to resume in as little as 60 days similar to that reported by Patzl et al. [10]. The distinct identification of reproductive status (estrous cycling and pregnancy) by non–invasive monitoring of fecal progestagens and estrogens suggest that these methods can be a valuable tool in the population management of captive giant anteaters to accurately time male and female introductions, diagnose pregnancy, and prepare for the impending birth of young.

Concentrations of fecal glucocorticoid (GC) metabolites in giant anteaters most paralleled those of estrogens during the estrous cycle and pregnancy. Active monitoring of GC metabolites, therefore, may aid husbandry managers determine sexual receptivity, pregnancy status, and impending parturition in this species. A similar elevation of GC metabolite concentrations were observed during the peri–estrus period in the giant panda and presumed to relate to an interaction with the estrogens as a result of changes in behavior, metabolic activity, and reproductive physiology [18]. The elevated concentration of GC metabolites observed during late pregnancy in the giant anteater were similar to those reported in the three banded armadillo (*Tolypeutes matacus)*, Belding’s ground squirrel (*Spermophilus belgingi)*, domestic cattle (*Bos Taurus)*, and the golden lion tamarin (*Leontopitecus rosalia)*[34,36,37]. An increased concentration of GC metabolites during late pregnancy in domestic livestock and humans is produced by the fetus and results in a greater enzymatic conversion of progesterone to estrogens, thus initiating parturition [34]. Our data suggest that similar physiological processes are likely to occur in the giant anteater. Interestingly, 2 of the 3 pregnant females examined in this study had the highest glucocorticoid concentrations during the presumed implantation period (secondary rise of P4) and then again within six days of the birth of young. Therefore, glucocorticoids may play a role in both implantation and parturition, and/or act in response to these acute stressful events. These findings provide baseline information regarding the role of fecal glucocorticoid concentrations during the estrous cycle and pregnancy that may be useful in future studies of reproductive and stress physiology in this and related species.

## Conclusions

This study confirmed through the examination of reproductive endocrinology that sexual maturity in giant anteaters can occur in as little as 1.8 years. Estrous cycles occurred year–round, but were shorter and more intermittent in younger nulliparous animals compared to a multiparous female. Gestation ranged 171–183 days, and pregnancy was characterized by a prolonged luteal phase and greater concentration of fecal P4 compared to the pattern of fecal P4 observed during the non-pregnant luteal phase. The pronounced elevation in P4, estrogen, and GC metabolites did not occur until late gestation after an initial post-mating delay ranging 81–105 days. This steroid hormone pattern is consistent with a period of delayed implantation that has not been previously described in giant anteaters. This study also indicates that GC metabolites are a useful marker of impending parturition as 2 of 3 pregnant females had the highest concentrations six days prior to the birth of young. Extraction and assay methodology are provided in this study as a guide for captive managers in the use of fecal hormone analyses to non-invasively monitor reproductive status in giant anteaters. However, differences in estrous cycle characteristics with age, and the protracted duration of pregnancy, must be considered to best improve breeding success and neonatal survival. For example, fecal P4 concentrations can be examined weekly or biweekly after observed mating. If P4 remains elevated for more than 40 days, pregnancy is likely and males should be kept separated from females. Pregnancy diagnosis can be further verified if a pronounced elevation of P4 (7-13x greater than luteal P4 concentrations) occurs for a period of 81–105 days after mating, and accompanied by elevated concentrations of estrogens and GCs indicative of late gestation. This study improves knowledge of the reproductive endocrinology of giant anteaters; however, several gaps in knowledge of their reproductive physiology remain. Future research directions include further studies on the delayed implantation strategy, ovulatory cues and sexual maturation, as well as investigation into male reproductive physiology and behavior for both captive and wild populations.

## Abbreviations

BSA: Bovine serum albumin; EIA: Enzyme immunoassay; E1: Estrone-glucuronide; E2: Estradiol; GC: Glucocorticoids; P4: Progestagens; SEM: Standard error of the mean; SD: Standard deviation.

## Competing interests

The authors declare that they have no competing interests.

## Authors’ contributions

KK carried out the extraction and immunoassay methods, analyzed the data, and wrote the manuscript, BM carried out the extraction and immunoassay methods, helped draft the manuscript, and revised the manuscript critically for important intellectual content, and MM carried out the extraction and immunoassay methods. All other authors made substantial contributions to conception, design, and acquisition of data. All authors read and approved the final manuscript.

## Supplementary Material

Additional file 1: Figure S1 Comparison of four extraction methods in five randomly selected fecal samples from giant anteater. (A) P4 metabolites, (B) E1 metabolites, and (C) E2 metabolites. Click here for file

Additional file 2: Figure S2 Validation of enzyme-immunoassays. Parallelism between standards and extracts of giant anteater feces for (A) progestagens, P4, (B) estrone-3-glucuronide, E1, (C) estradiol-17β, E2 and (D) glucocorticoid, GC, metabolites as determined by enzyme immunoassay (see Methods). Click here for file
