# Supplementary figures and images for: Fecal estrogen, progestagen and glucocorticoid metabolites during the estrous cycle and pregnancy in the giant anteater (Myrmecophaga tridactyla): evidence for delayed implantation
Source: Reprod Biol Endocrinol. 2013 Aug 27;11:83. doi: 10.1186/1477-7827-11-83 (PMC3765926; doi:10.1186/1477-7827-11-83)

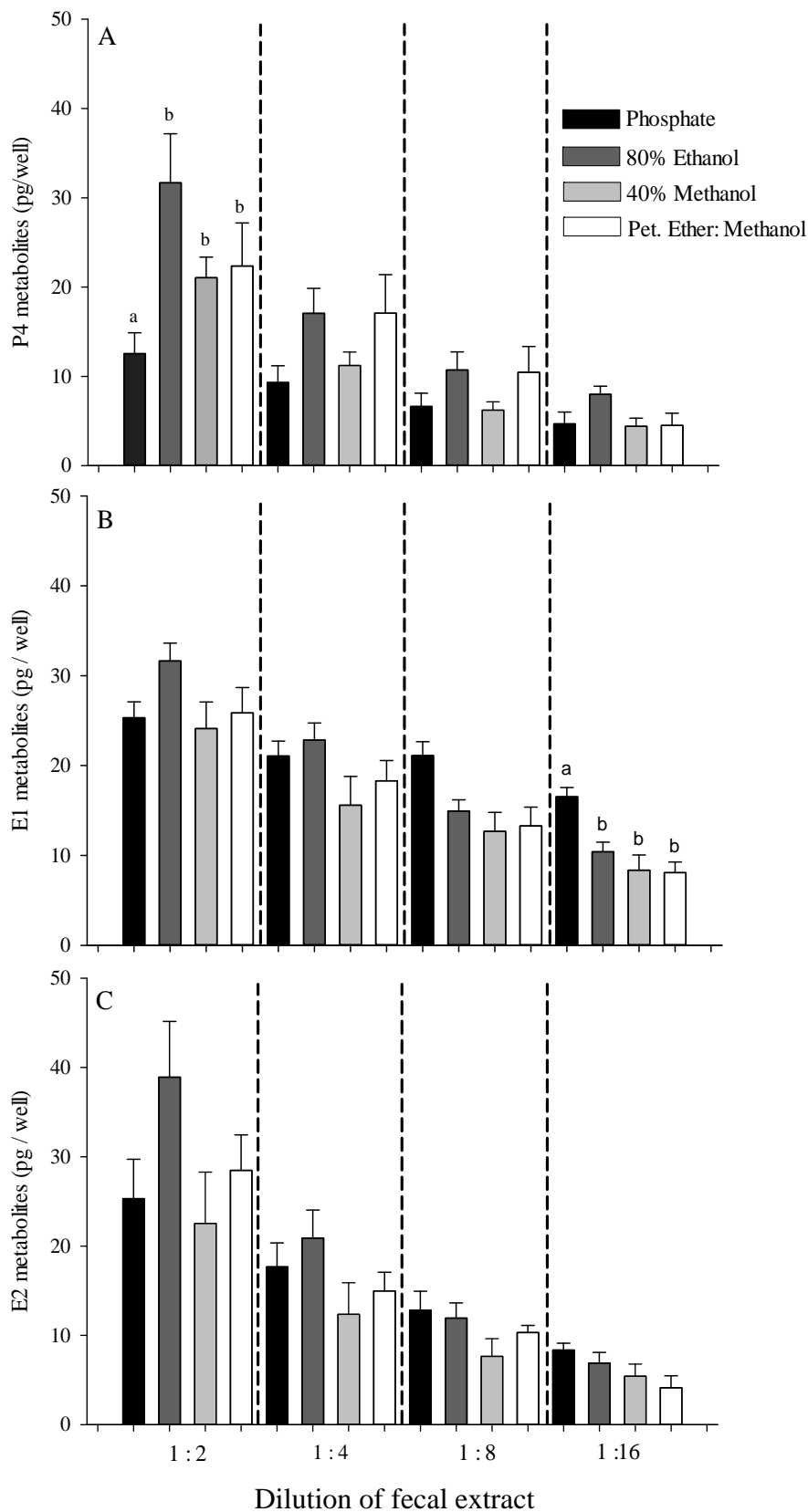

Supplement: Additional file 1: Figure S1 — Comparison of four extraction methods in five randomly selected fecal samples from giant anteater. (A) P4 metabolites, (B) E1 metabolites, and (C) E2 metabolites. [file 1477-7827-11-83-S1.pdf]

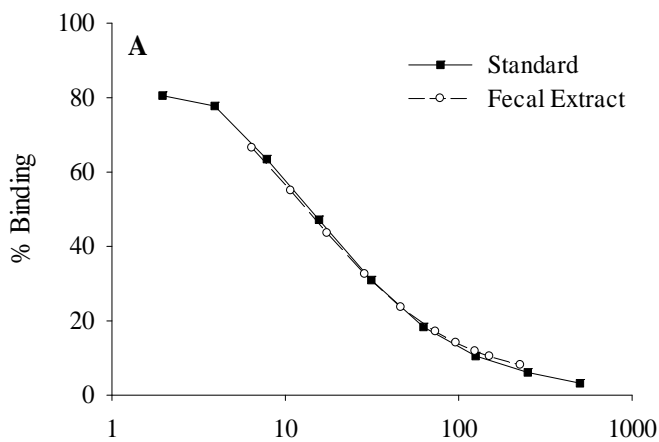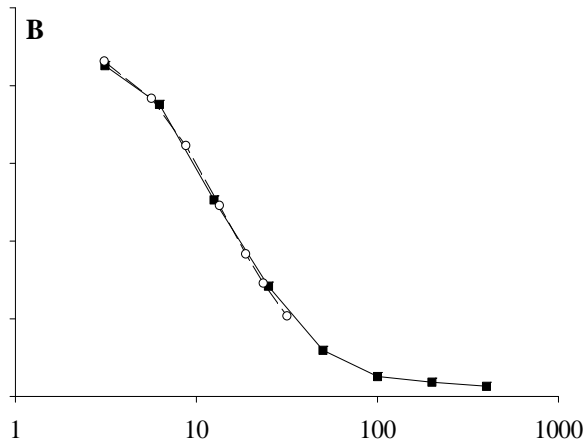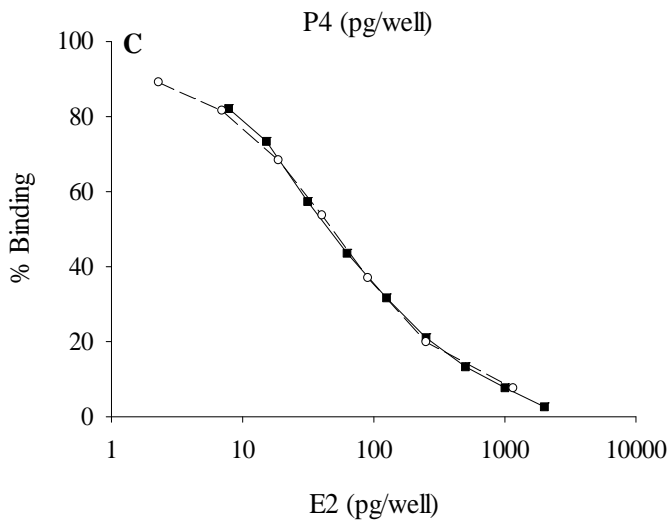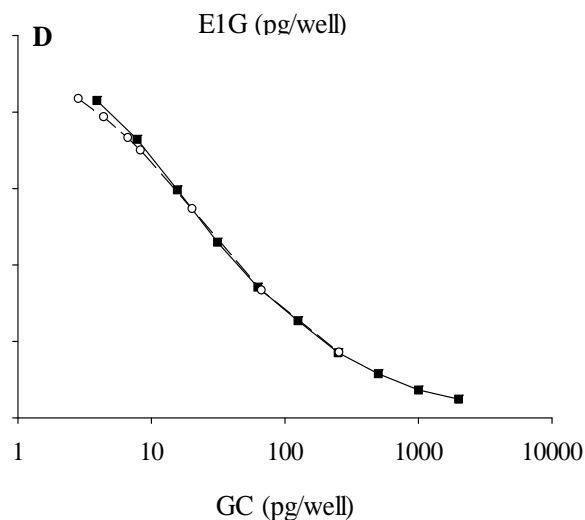

Supplement: Additional file 2: Figure S2 — Validation of enzyme-immunoassays. Parallelism between standards and extracts of giant anteater feces for (A) progestagens, P4, (B) estrone-3-glucuronide, E1, (C) estradiol-17β, E2 and (D) glucocorticoid, GC, metabolites as determined by enzyme immunoassay (see Methods). [file 1477-7827-11-83-S2.pdf]
